# Randomized Trial Comparing the Electronic Composite Psychosocial Screener YouthCHAT With a Clinician-Interview Assessment for Young People: A Study Protocol

**DOI:** 10.2196/resprot.7995

**Published:** 2017-07-31

**Authors:** Hiran Thabrew, Arden Corter, Felicity Goodyear-Smith, Mary Goldfinch

**Affiliations:** ^1^ University of Auckland Auckland New Zealand; ^2^ Tamaki College Auckland New Zealand

**Keywords:** mass screening, adolescents, substance-related disorders, depression, anxiety, primary health care, school health, services, chronic disease

## Abstract

**Background:**

Psychosocial problems such as depression, anxiety, and substance abuse are common and burdensome in young people, particularly those with long-term physical conditions such as asthma and diabetes. In New Zealand, “screening” for such problems is undertaken routinely only with Year 9 students in low-decile schools and opportunistically in pediatric settings using a nonvalidated and time-consuming clinician-administered Home, Education/employment, Eating, Activity, Drugs, Sexuality, Suicide/depression, Safety (HEEADSSS) interview. The Youth version, Case-finding and Help Assessment Tool (YouthCHAT) is a relatively new, locally developed, eTablet-based composite screener for identifying similar psychosocial issues to HEEADSSS. Based on individually validated screening instruments, it is self-administered within minutes. Preliminary testing has revealed its acceptability to young people, but further research is required to expand its modules to cover all HEEADSSS domains, to evaluate its acceptability for young people with and without long-term physical conditions, and to compare its effectiveness against HEEADSSS.

**Objective:**

Our aim is to (1) ascertain acceptability and utility of YouthCHAT for children with long-term physical illness and high school students, (2) validate three additional YouthCHAT domains against comparable HEEADSSS domains, and (3) compare the performance of YouthCHAT and HEEADSSS in the high school setting.

**Methods:**

During the first phase of the study, three additional YouthCHAT domains were codesigned with high school students. During the second phase of the study, the updated version of YouthCHAT will be administered to 30 young people with long-term physical conditions, and to 150 high school students either before or after HEEADSSS in the form of a randomized trial with counter-balanced design. Primary outcomes include comparability between HEEADSSS and YouthCHAT in detecting psychosocial issues, and time to administer; acceptability of YouthCHAT as an acceptable alternative or companion to HEEADSSS assessment; and the utility of YouthCHAT in helping streamline assessment processes.

**Results:**

Recruitment for the first phase of this project commenced in November 2016, and the phase will run from February to November 2017.

**Conclusions:**

If YouthCHAT is found to be acceptable to study participants and as effective as a HEEADSSS assessment, it could be an innovative and more efficient means of routine screening for common psychosocial health issues in young people with and without long-term physical conditions.

**Trial Registration:**

Australian New Zealand Clinical Trials Registry (ANZCTR) ACTRN12616001243404p; https://www.anzctr.org.au/Trial/Registration/TrialReview.aspx?id=371422 (Archived by WebCite at http://www.webcitation.org/ 6rmlEiM1L)

## Introduction

Psychosocial problems are a significant issue for young people in New Zealand, particularly those with long-term physical conditions. Recent figures indicate that 27% of high school students are affected by anxiety and depression [1], and at 18 years of age the prevalence of hazardous drinking exceeds 50%. New Zealand’s 2011 youth suicide rate was the second highest in the Organisation for Economic Co-operation and Development (OECD) [2]. As mental health and lifestyle issues often present in adolescence, it is important to intervene early so that problems do not become entrenched, leading to costly poor health and social outcomes [3-5].

Young people with long-term physical conditions are at even greater risk of psychosocial problems [6]. Depressive symptoms have been reported in as many as 40% of children with a long-term condition and socialization problems [7]. Anxiety has also been identified in children and young people with long-term physical conditions as an area of clinical significance [8]. The likelihood of such problems developing is related to young people’s internal ability to manage stress, which develops over time; family factors; and the impact of illness and treatment, particularly association with distress and pain [9].

The World Health Organization and New Zealand policies and programs all emphasize the importance of addressing youth mental health with appropriately targeted services and recognize the need for easily accessible services, including use of appropriate tools [10-12]. Young people want a greater say in how services are designed and delivered, and they expect services to be diverse, contemporary, and responsive. These requirements are echoed in many national policies including the New Zealand Mental Health Commission’s Blueprint II [13,14], the New Zealand Ministry of Health’s Rising to the Challenge 2012-2017 [15], Statement of Intent 2014-2018 [16], and the New Zealand Suicide Prevention Action Plan 2013-2016 [17].

Home, Education/employment, Eating, Activities, Drugs and Alcohol, Sexuality, Suicide/depression, Safety (HEEADSSS) assessment is a clinician-administered, interview-based assessment of youth that can identify mental health and substance use problems [18,19]. Currently, all Year 9 (13-14 year olds) students in low-decile schools (those with the highest proportion of students from low socioeconomic communities), and some attendees at primary care and pediatric services in New Zealand are screened for well-being via HEEADSSS assessment. While HEEADSSS assessment offers a straightforward, holistic, and gradual approach to assessing young people across many domains, it is a face-to-face assessment, not a screening tool. Drawbacks include its lack of validation for problem identification, the cost of resourcing, time required for its administration (up to 45 minutes), and variability of assessment depending on the skill and experience of the assessor. In many high schools, it takes a full school year and more than one school nurse to screen all Year 9 students (personal communication M Goldfinch). In pediatric settings around New Zealand, routine screening for psychosocial problems is not usually undertaken and HEEADSSS assessments are conducted by some medical and nursing staff in an ad hoc manner.

The Youth version of the electronic Case-Finding and Help Assessment Tool (YouthCHAT) [20,21] is a self-report screening tool that covers ten domains: smoking, drinking, recreational drug use (based on the Substances and Choices Scale [SACS]) [22], problematic gambling, depression (based on the Patient Health Questionnaire – Adolescent Version [PHQ-A]) [23], anxiety (based on the Generalized Anxiety Disorder scale, 7 item [GAD-7]) [24], sexual health, exposure to abuse, anger management, and physical activity. For each positive domain screened, there is a “help” question that asks participants if they would “like help today,” “want help, but another time,” or “don’t want help.” Responses to the “help” question support conversations between young people and their health providers about the issues they would like addressed, which facilitates shared decision making, with increased likelihood that real sustained changes will be made.

YouthCHAT provides summary reports to authorized school/health clinic staff plus a guide to stepped-care management from self-help through to secondary care. It is a rapid screening system (taking 5-15 minutes to complete) and can be conducted on any electronic device. YouthCHAT has a 14-year history of development in primary care, youth, and school clinics [20,21,25-31]. Recently, it was successfully implemented in a rural school clinic and favorably received by both young patients and clinic staff. Evidence indicates that students are more likely to disclose information about challenges they are facing through electronic means than an initial in-person assessment [32].

As a validated and rapid screening tool, YouthCHAT has the potential to overcome barriers associated with HEEADSSS assessment, while providing a similar holistic assessment of mental health and lifestyle issues. Additionally, as YouthCHAT collects and provides electronic data, the additional paperwork that HEEADSSS requires is not needed. YouthCHAT may be a more efficient screener and serve as a useful substitute or precursor to HEEADSSS assessment. YouthCHAT currently screens for all HEEADSSS domains except three: eating issues, conduct problems/bullying, and resilience/strengths. Additional screening questions to cover these domains have been added, based on those available in the existing literature, with appropriate wording advised by high school students, including those of Māori and Pacific Island descent via focus groups to ensure the updated version of YouthCHAT is appropriate for young people from all major ethnic groups in New Zealand.

The aims of this study are to (1) validate three additional YouthCHAT domains against comparable HEEADSSS domains, (2) ascertain the acceptability and utility of YouthCHAT for children with long-term physical illness and high school students, and (3) compare the performance of YouthCHAT and HEEADSSS in the high school setting.

We hypothesize that a version of YouthCHAT matched to HEEADSSS for all domains will be an acceptable alternative to a HEEADSSS assessment to both young people and the clinical staff conducting the screen. We further hypothesize that screening using YouthCHAT, followed up by a targeted or full HEEADSSS assessment where indicated, will be a much more streamlined and efficient system to enable identification and intervention of young people at risk.

## Methods

### Study Design

We will use a mixed-methods participatory action research approach to development and validation of a YouthCHAT mapped to all HEEADSSS domains, and assessment of the acceptability and utility of YouthCHAT at a tertiary pediatric hospital and a low-decile high school. A randomized controlled trial (RCT) with counterbalanced design will be conducted at the high school to assess the comparability of YouthCHAT and HEEADSSS.

### Participants and Recruitment

During the first phase, up to 20 senior students aged 16-18 years from Tamaki College, a low-decile high school in Auckland, New Zealand, were invited to participate in focus groups to update YouthCHAT via flyers sent to their school. These students were chosen as they were all able to give their own consent.

During the second phase, approximately 150 Year 9 students (aged 13-14 years) from the same high school will be invited to participate in the RCT and school-based acceptability evaluation via letters will be sent to their parents. Parents may use a reply-paid form to opt out of the study if they do not wish their children to participate. However, regardless of this, all students will receive mandatory HEEADSSS assessments (as is routine for all students in this class). Students who choose to participate in the study will be randomly assigned to one of the two conditions (YouthCHAT followed by HEEADSSS assessment or HEEADSSS assessment followed by YouthCHAT) via a computer-generated random numbers table, with resulting lists provided to clinic staff. Sampling will be consecutive until all enrolled students have completed assessment. All screening will take place in the student health clinic on a desktop computer. A subset of 32 students will be invited to participate in four focus groups, and four school staff (nurses and counsellors) will be invited to complete semistructured interviews on the acceptability, utility, and comparability of YouthCHAT and HEEADSSS assessment. This age group of participants was chosen as it matches the age group of students who routinely receive HEEADSSS assessments in New Zealand low-decile schools.

Also during the second phase, 30 young people aged 13-18 years with long-term physical conditions (eg, diabetes, asthma, cystic fibrosis and congenital heart disease) attending outpatient clinics at Starship Children’s Hospital, a tertiary pediatric hospital will be invited to participate in the study via flyers disseminated by clinic staff. Following individual or parental consent (and personal assent), they will be asked to complete YouthCHAT and a postconsultation survey to assess its acceptability and utility. This age group of participants has been chosen as it matches the age range to which YouthCHAT is targeted. Pediatricians and specialist nurses who have conducted these consultations will be also invited to undertake a short semistructured interview to provide a clinical perspective on the use of YouthCHAT in a pediatric setting.

### Outcome Variables

The main study variables are (1) comparability (eg, is YouthCHAT at least as good or better than HEEADSSS assessment at detecting mental health and lifestyle issues among Year 9 youth? Is it a faster method of screening?), (2) acceptability (eg, is YouthCHAT content/format an acceptable alternative or companion to HEEADSSS assessment?), and (3) utility (eg, is YouthCHAT useful in helping to streamline assessment processes?).

Our primary outcome measures are (1) comparable detection rates for each domain for HEADSSS and YouthCHAT as measured by mental health or lifestyle issue “present” or “absent” (Tamaki sample only), (2) time taken to complete YouthCHAT and HEEADSSS screening (Tamaki sample only), and (3) acceptability and utility of YouthCHAT as assessed via feedback from staff and students/patients (Starship and Tamaki participants). Secondary outcome measures include differences in YouthCHAT versus HEEADSSS screening outcomes by gender and ethnicity.

### Measures and Data Collection

#### YouthCHAT

YouthCHAT covers 10 domains: smoking, drinking, recreational drug use, problematic gambling, depression, anxiety, sexual health, exposure to abuse, anger management, and physical activity. In the case of positive screens, branching logic links users to validated assessment tools including SACS for drug and alcohol use, PHQ-9 for depression, and GAD-7 for anxiety. For the purpose of this project, three new domains have been added to YouthCHAT related to diet, body image, and bullying. Questions related to these domains have been developed based on existing validated tools and have been trialled with student focus groups prior to initiation of the study comparing YouthCHAT with HEEADSSS. Interested readers are welcome to contact the authors for the complete list of YouthCHAT questions.

#### HEEADSSS

HEEADSSS is a clinician-administered, interview-based assessment of youth that can identify mental health and substance use problems [18,19]. It covers the domains of Home, Education/employment, Eating, Activities, Drugs and Alcohol, Sexuality, Suicide/depression, and Safety. It is currently administered by trained school health nurses to all Year 9 students in low-decile schools and some attendees at primary care and pediatric services in New Zealand.

#### Administrative Data

Administrative data such as staff time to complete screening/assessments, psychosocial issues identified via HEEADSSS and YouthCHAT, and referrals made based on positive screens will be collected.

#### Participant Survey

Following completion of YouthCHAT, participants at Starship and Tamaki College will be asked to complete a survey comprised of Likert scales, free-text options, and forced-choice questions on the acceptability and utility of YouthCHAT.

#### Clinician Interviews

Taped and transcribed semistructured interviews with the participating school, Starship, and hospital staff will be conducted (N=10-15). Interviews will focus on perceptions of the utility and acceptability of YouthCHAT (both settings) and comparability with HEEADSSS (Tamaki only) and will be conducted in person or by telephone as preferred by participants.

#### Focus Groups

Two taped and transcribed student focus groups to advise on the face validity and wording of the newly added YouthCHAT modules were conducted at Tamaki College during the first phase of the research. An additional four taped and transcribed student focus groups that address acceptability and utility of the assessments will be conducted at Tamaki College at the end of the school year during the second phase.

### Procedure

During the first phase, screening questions to address the HEEADSSS domains of eating issues, conduct problems/bullying, and resilience/strengths were selected based on existing validated tools sourced through the peer-reviewed literature. These questions were adapted to the New Zealand youth context using focus groups with high school students. Students were asked to judge whether the questions make sense, explain what they think the questions are asking, identify any words or phrases they find unclear, and suggest alternative youth-friendly language. They were also asked to indicate the order in which the questions should appear and enhancements followed majority opinion. In the spirit of biculturalism, at least one focus group was undertaken with only Māori young people to identify any culturally relevant viewpoints, issues, or concerns. Focus groups were audiotaped, confidentially transcribed, and suggestions categorized and tabulated. The resulting additional modules were programmed into YouthCHAT.

During the second phase, a counterbalanced RCT of YouthCHAT and HEEADSSS will be undertaken with high school students. During this study, one group will receive YouthCHAT before HEEADSSS assessment (Condition 1), and the other HEEADSSS assessment before YouthCHAT (Condition 2) and findings from YouthCHAT and HEEADSSS assessment will be compared. In addition, the acceptability and utility of YouthCHAT will be evaluated in two settings—the high school where YouthCHAT and HEEADSSS assessments are undertaken and a tertiary pediatric hospital setting where young people aged 13-18 years with long-term physical conditions are attending pediatric outpatient appointments. Following the use of YouthCHAT by both audiences, focus groups will be undertaken to identify positive feedback, issues, and concerns regarding the electronic screener. Once again, at least one focus group will be undertaken in each setting with only Māori young people to identify any culturally relevant viewpoints, issues, or concerns. In addition to feedback from young people, feedback regarding appeal and utility will be sourced from school nurses at the high school and pediatricians and specialist nurses at the pediatric hospital.

Any focus group participants at the tertiary pediatric hospital who are distressed or develop psychosocial concerns during or following the study will be referred to the hospital-based consult liaison (mental health) service for further assessment and management, in a similar manner to what would occur as part of usual care. Within the high school setting, stepped-care resources and a clinical pathway (algorithms relating to domain and severity of identified problem) will be provided to augment usual care options. These include self-help resources (helplines, psychoeducation and information sheets, websites, and e-therapies), clinician interventions (medications and brief interventions), local community agencies, and referral to adolescent secondary care mental health and drug and alcohol services.

### Data Handling

HealthLink, an electronic data management system, will provide secure storage of all YouthCHAT screening data. It will also enable encrypted secure data transmission to the clinic’s MedTech32 practice management system (PMS) software by the same private network used for their electronic referrals solution. Clinical staff will access their YouthCHAT summary reports directly from the website. This allows a brief version to be cut and pasted into clinical notes, or a PDF generated to attach to the patient file. HEEADSSS data will be managed as per standard care via the school nurse and his/her team. All HEEADSSS data are entered into the PMS, with a summary report provided to the Ministry of Health. HEEADSSS data will need to be manually inputted into a Microsoft Excel spreadsheet by a research assistant. Each YouthCHAT generates a unique code for each participant. This code will be transferred by the school nurse into the PMS HEEADSSS file for data-matching. Thus, research data will be anonymized and de-identified prior to being analyzed.

### Power Calculation and Data Analysis

For the RCT component of the study, an estimated 150 Year 9 students will be randomly allocated to either Condition 1 (YouthCHAT first) or Condition 2 (HEEADSSS first). A power calculation shows that given 144 participants, there will be 90% power to detect differences between the two assessments assuming a mean duration of 15 minutes for YouthCHAT (SD 30) and 45 minutes for HEEADSSS assessment (SD 90).

Quantitative data will be analyzed using Microsoft Excel and Statistical Software Package (SPSS). Analyses will include basic descriptive statistics (eg, number of youth screened, YouthCHAT summary data, HEEADSSS summary data, demographic characteristics of the sample). Parametric data will be analyzed using *t* tests and analysis of variance (ANOVA). Nonparametric data will be analyzed using Wilcoxon signed-rank tests.

Qualitative data will be analyzed using a general inductive approach [33] with collated text analyzed to identify emerging themes, which will then be independently coded by 2 researchers with consensus reached by adjudication. Data analysis will be conducted at the end of 2017.

### Ethical Approval

This trial received ethical approval from the New Zealand Health and Disability Ethics Committee (Reference: 16/CEN/137) on October 14, 2016.

## Results

Participant recruitment for the first phase of this study was undertaken in November 2016. Recruitment for the second phase of the study commenced in January 2017 and will continue until November 2017, with results expected before July 2018.

## Discussion

### Principal Considerations

If effective, YouthCHAT could offer an inexpensive, simple, scientifically valid, and acceptable vehicle for routine psychosocial screening in all young people in New Zealand high schools (not just those in Year 9 within low-decile schools) and all young people with long-term physical conditions (eg, during routine outpatient visits/annual check-up appointments in pediatric settings). It may also be useful to screen young people in multiple settings overseas.

On an individual level, early detection and intervention of mental health and substance misuse issues can have downstream benefits including improved physical health and better compliance with medical treatments, better engagement in education and subsequent employment, reduced youth suicide, and improved social relationships. Detecting and intervening in risky sexual behaviors can lead to a reduction in unwanted pregnancies and sexually transmitted infections. Dealing with bullying, physical and sexual abuse, and assisting youth with anger control will likely have significant psychosocial benefits. Helping youth make healthy eating choices and increase their physical activity can contribute to their physical and mental health and well-being, and reduce the incidence of conditions such as obesity and related illnesses. Although one other HEEADSSS-based electronic screener for young people (TickIT) has been evaluated for acceptability with this audience [34], no composite screener of this kind has yet been shown to be as effective in identifying psychosocial problems as face-to-face HEEADSSS assessment and there is no other gold standard for psychosocial screening. Evidence of efficacy of screening instruments is as relevant as evidence of acceptability, and this study hopes to demonstrate both for YouthCHAT.

On a population level, the potential impact of such screening and early intervention is enormous. HEEADSSS assessment is currently costly and is therefore limited to a subset of the adolescent population of New Zealand. No routine psychosocial screening is currently undertaken with young people who have long-term physical conditions and are at higher risk of such problems. If the updated version of YouthCHAT is as effective as HEEADSSS assessment in detecting psychosocial issue and is as acceptable to young people, it may be routinely introduced as a first-line screener for psychosocial problems in all youth, especially those at high risk of psychosocial problems. This is likely to have cost benefits with short-term economic impacts including reductions in staff time required to conduct HEEADSSS assessments. Long term, there is the potential for cost effectiveness across many spheres, such as reductions in health care costs associated with helping youth maintain better health through lifestyle choices, reductions in benefits payouts via improving chances of education and employment, and reductions in costs associated with the youth justice system via supporting youth to make prosocial lifestyle choices.

A key advantage of YouthCHAT is its person-centered approach. Young people are asked to indicate which issues they would like help with. Clinical staff are provided with stepped-care resources to use depending on the issue and its severity, including a number of self-management options such as e-therapies. The systematic approach of annual screening and provision of algorithms for stepped-care intervention is likely to lead to early and more comprehensive intervention of youth mental health, substance misuse, and other lifestyle issues, which will have large impacts on their subsequent physical, mental, and social health and well-being, as outlined above.

### Conclusion and Recommendations

If the outcomes indicate that YouthCHAT provides a comparable mental health and psychosocial assessment to HEEADDSSS, is acceptable to young people and clinical staff, and is faster to administer, we propose the following clinical changes.

- All Year 9 students in low-decile high schools should be screened using YouthCHAT, and currently routine HEEADSSS assessments should not be undertaken where there is a negative screen. Instead, a tailored (targeted) HEEADSSS assessment should be conducted for the YouthCHAT positive domains.
- Young people with long-term physical conditions who are admitted to pediatric hospitals or attending routine outpatient clinics for long-term physical conditions should undergo an annual YouthCHAT screen, with targeted HEEADSSS assessment where needed.
- All students in New Zealand high schools should be screened using YouthCHAT annually.
- The potential for YouthCHAT to be used in other countries should also be explored.

## Abbreviations

GAD-7: Generalized Anxiety Disorder scale – 7 item
HEEADSSS: Home, Education/employment, Activity, Drugs, Sexuality, Suicide/depression, Safety
MS: Practice Management System
PHQ-A: Patient Health Questionnaire – Adolescent version
RCT: randomized controlled trial
SACS: Substances and Choices Scale
YouthCHAT: Youth version ‒ Case-finding and Help Assessment Tool
